# Room temperature ferroelectricity in fluoroperovskite thin films

**DOI:** 10.1038/s41598-017-07834-0

**Published:** 2017-08-03

**Authors:** Ming Yang, Amit KC, A. C. Garcia-Castro, Pavel Borisov, E. Bousquet, David Lederman, Aldo H. Romero, Cheng Cen

**Affiliations:** 1 Department of Physics and Astronomy, West Virginia University, Morgantown, West Virginia 26506 USA; 2 Department of Physics, University of California, Santa Cruz, California 95064 USA; 3 Department of Physics, School of Science, Loughborough University, Loughborough, LE11 3TU UK; 4 Physique Théorique des Matériaux, Université de Liège, B-4000 Sart-Tilman, Belgium

## Abstract

The NaMnF_3_ fluoride-perovskite has been found, theoretically, to be ferroelectric under epitaxial strain becoming a promising alternative to conventional oxides for multiferroic applications. Nevertheless, this fluoroperovskite has not been experimentally verified to be ferroelectric so far. Here we report signatures of room temperature ferroelectricity observed in perovskite NaMnF_3_ thin films grown on SrTiO_3_. Using piezoresponse force microscopy, we studied the evolution of ferroelectric polarization in response to external and built-in electric fields. Density functional theory calculations were also performed to help understand the strong competition between ferroelectric and paraelectric phases as well as the profound influences of strain. These results, together with the magnetic order previously reported in the same material, pave the way to future multiferroic and magnetoelectric investigations in fluoroperovskites.

## Introduction

Magnetoelectric materials, which allow manipulations of magnetic (electric) polarization by electric (magnetic) field, are intensively sought after. A closely related class of materials is composed of multiferroics^1–3^, where multiple ferroic orders (typically ferroelectricity and ferromagnetism/antiferromagnetism) coexist, but the coupling between them may or may not be present. Many transition metal oxides with perovskite structures are multiferroic, such as BiFeO_3_^4, 5^, YMnO_3_^6, 7^, and TbMnO_3_^8, 9^. So far, magnetoelectric applications of these materials are limited by weak coupling between the ferroelectric and antiferromagnetic orders and/or by weak electric/magnetic polarizations^10^.

Besides the well-studied oxides, other materials with possible magneto-electric coupling are under investigations as well, as in the case of fluoride materials^11–15^. In particular, recent calculations predicted multiferroic signatures in the perovskite fluoride NaMnF_3_^16^. In this compound, geometric effects from the displacements of Na cations are expected to generate a ferroelectric instability under strain leading to a stable polar ground state where the latter instability is condensed. Additionally, presence of spin-canting in the ground antiferromagnetic phase was also predicted for this material. The resultant weak ferromagnetic component could become useful to tune the polarization by an external field. Recently, quasi-epitaxial thin films of NaMnF_3_ on SrTiO_3_ substrates were successfully grown by molecular beam epitaxy (MBE), in which the low temperature antiferromagnetic order and spin-canting induced magnetization were verified experimentally^17^. Remarkably, only a few fluoroperovskites, such as CsPbF_3_^18, 19^ and NaCaF_3_^20^, have shown ferroelectricity so far. Though none of them contains a magnetically active cation. Therefore, the confirmation of the ferroelectric state in NaMnF_3_ is of high importance in the multiferroics field since it would be the first perovskite-like fluoride to exhibit a multiferroic behavior. We note that, in films grown on a conducting SrRuO_3_ epi-layers pre-deposited on SrTiO_3_, temperature dependent dielectric measurements showed signs of an onset of low-temperature ferroelectric order-disorder transition, but long range ferroelectric order was not observed above 10 K^17^. For films grown without the SrRuO_3_ back contact layer, similar measurements were not possible, and the ferroelectric properties of these films need to be evaluated by other methods.

Here we report on the ferroelectric properties of NaMnF_3_ films grown directly on SrTiO_3_ substrates by piezoresponse force microscopy (PFM). A preferred polarization pointing out of the plane was found in the as-grown state. Repeatable ferroelectric switching by biased scanning probe was observed at room temperature. An interesting 180° out-of-plane polarization flip by the application of an in-plane electric field was discovered as well. The PFM results are consistent with the weak ferroelectricity revealed by DFT calculations. At low temperatures, we also discovered a tunable zero-bias photocurrent that was attributed to the persistent polarizations in NaMnF_3_. The collection of experiments not only provides evidences of room temperature ferroelectricity, but also suggests the significant impacts of the electric boundary conditions and strain on the ferroelectric states.

## Results

### DFT calculations of the ferroelectric and paraelectric phases in NaMnF_3_

**Figure 1:**
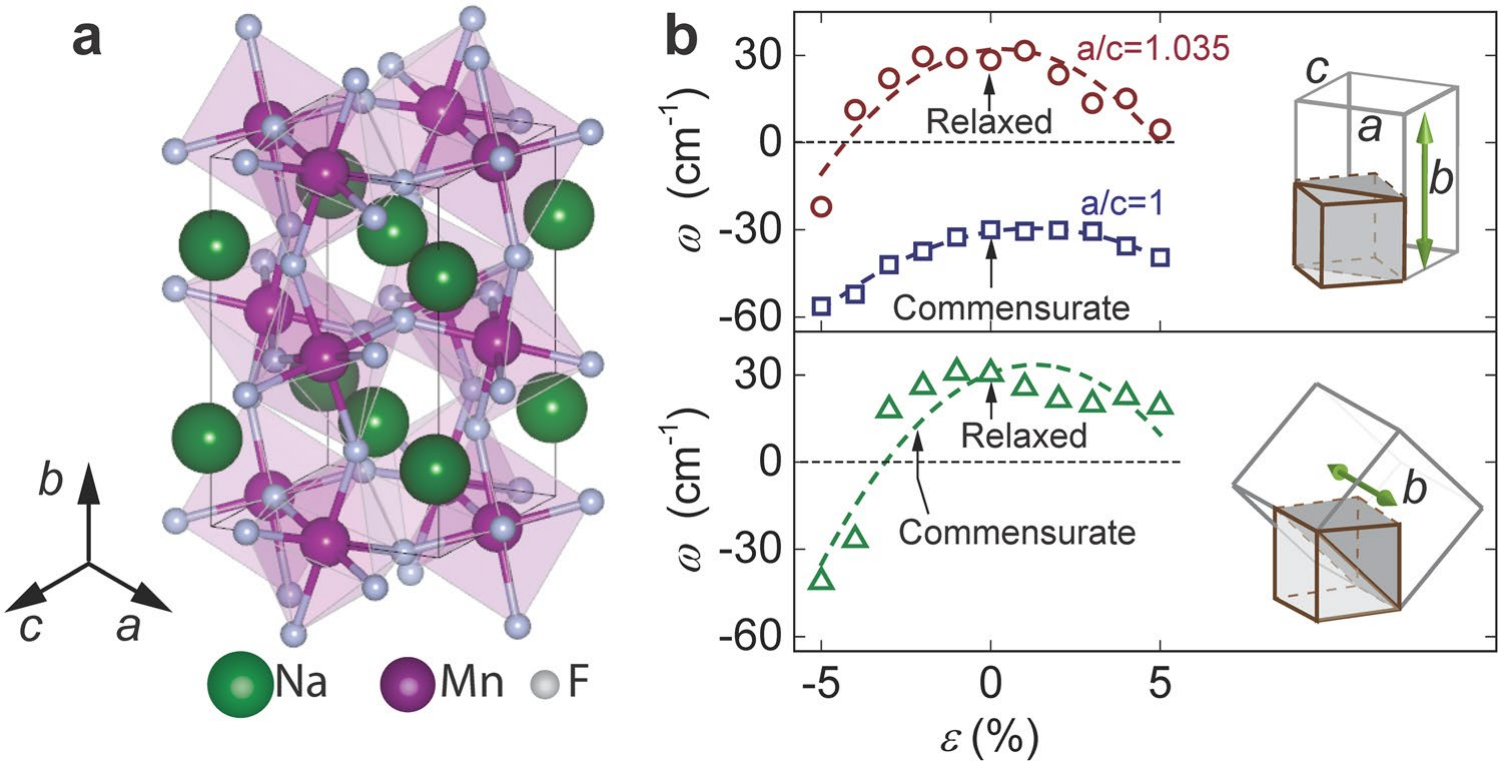
Ferroelectricity depending on growth and strain configurations. (**a**) Schematic of NaMnF_3_ unit cell^21^. (**b**) Polar mode frequency as functions of isotropic strain in the plane parallel to SrTiO_3_ (001) surface when NaMnF_3_ is grown with b-axis out-of-plane (top, the two curves indicate different a/c ratios) or in-plane (bottom). Arrows indicate the data points corresponding to commensurate growth and completely relaxed cases. Dashed lines are added as guide for the eye. Insets illustrate the orientations of NaMnF_3_ lattice relative to the cubic SrTiO_3_ unit cell.

NaMnF_3_ thin films were grown on SrTiO_3_ (001) substrates by molecular beam epitaxy (MBE)^17^. Bulk NaMnF_3_ at room temperature has an orthorhombically distorted perovskite structure with lattice parameters *a* = 5.757 Å, *b* = 8.008 Å, *c* = 5.548 Å (Fig. 1a)^22^. When grown on the (001) surface of SrTiO_3_ (*a*_STO_ = 3.905 Å), there are two configurations with best lattice matching: one with the longest orthorhombic axis (*b*-axis) out-of-plane and the *ac*-plane rotated 45° relative to the cubic cell of SrTiO_3_ (Fig. 1b top), and the other one with the *b*-axis in-plane (Fig. 1b bottom). The small lattice mismatches in both cases favor a compressive strain at the film-substrate interface.

Density functional theory (DFT)^23, 24^ calculations were performed to evaluate the ferroelectricity of NaMnF_3_ films under different strain and growth configurations. The calculated polar mode frequencies in the lowest-energy paraelectric phases are shown in Fig. 1b. Imaginary frequencies, indicating that the paraelectric phase is no longer stable, are reported as negative values for clarity.

In the growth configuration with *b*-axis out-of-plane, the two lowest-energy phases are the paraelectric *Pnma* phase and the ferroelectric *Pna2*_*1*_ phase with polar axis along the *b*-axis. For completely relaxed films (Fig. 1b, top graph, circle marked by arrow), the paraelectric *Pnma* phase is the stable ground state (Δ*E* = *E*_*Pna21*_ − *E*_*Pnma*_ = 24.84 meV/unit cell (meV/uc)). When isotropic in-plane strain ($\varepsilon$) is applied, values of *a* and *c* change proportionally keeping their ratio of $a/c\approx 1.035$ constant. The film remains paraelectric under small isotropic strains, and only becomes ferroelectric when compressive strain larger than −4% is applied (Fig. 1b, top graph, circles). Commensurate growth relation with SrTiO_3_ requires an anisotropic in-plane strain that reduces the *a/c* ratio to 1. In this case, NaMnF_3_ unit cell remains orthorhombic due to the octahedral rotations that break the four-fold symmetry. However, the polar soft-mode is no longer vibrationally stable and the ferroelectric *Pna2*_*1*_ phase becomes the ground state (Δ*E* = −4.21 meV/uc at *ε = *0%, Fig. 1b, top graph, square marked by arrow). This ferroelectric state at $a/c=1$ is robust against isotropic in-plane strain (Fig. 1b, top graph, squares). This indicates that small reductions of $a/c$ ratio can profoundly influence the generation of ferroelectricity, which is likely caused by the small size of Na atoms and the resultant geometric nature of the ferroelectric ordering^15^.

When NaMnF_3_ is grown with *b*-axis parallel to the SrTiO_3_ (001) surface, even the application of a very small in-plane strain will change the unit cell into a monoclinic symmetry. The structure with small strain is in the paraelectric *P2*_*1*_ */m* phase (Fig. 1b, bottom graph). At an isotropic in-plane compressive strain of 2.3%, commensurate growth relation with SrTiO_3_ can be reached, which still corresponds to a paraelectric phase. The polar mode frequency only becomes imaginary when the in-plane compressive strain exceeds −3% (Fig. 1b, bottom graph). In this case, NaMnF_3_ transits into a ferroelectric *P2*_*1*_ phase (Δ*E* = ${E}_{P{2}_{1}}-{E}_{Pnma}$ = −4.06 meV/uc at *ε* = −4%) in which the polar axis is also along the *b*-axis.

X-ray diffraction (XRD) results reported in ref. 17 show that the MBE grown films are mostly relaxed and domains with *b-*axis in-plane and out-of-plane are both present. Therefore, the majority portions of the films are likely paraelectric. However, the variation of lattice parameters detected by XRD ($a/c=1.034\pm 0.017$) for the out-of-plane domains suggests the possible existence of regions with *a/c* ratios closer to one. In these regions, when a slight compressive strain is introduced, which in practice could be produced near the interface and grain boundaries or by surface adsorption and defects, the ferroelectric *Pna2*_*1*_ phase will become energetically favorable. When compared to the robust ferroelectricity predicted for the fully commensurate growth, however, the ferroelectric polarization in these regions is likely more easily affected by experimental perturbations.

### PFM characterizations of as-grown film

Two 200 μm × 200 μm gold electrodes with a 40 μm gap were deposited on the sample surfaces by photolithography and electron-beam evaporation. Here we show PFM measurements performed on a sample with 50 nm NaMnF_3_ at room temperature in an “out-of-plane” configuration where the flexural deflections of the cantilever were monitored. During PFM measurements, the sample was held at ground potential through the surface electrodes and an AC excitation voltage (*V*_AC_) with frequency *f* was applied to the PFM probe. The amplitude and phase of the out-of-plane deformation at the sample surface in response to *V*_AC_ were lock-in detected.

**Figure 2:**
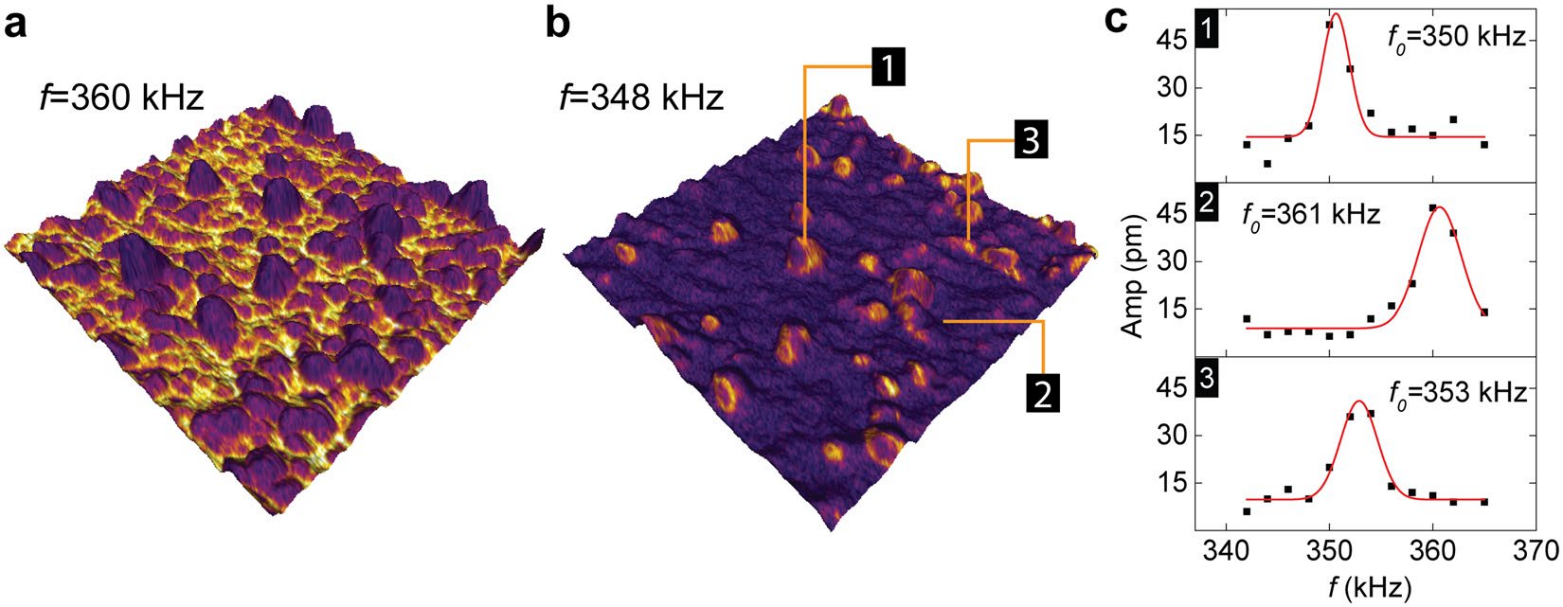
PFM performed on NaMnF_3_ thin film. (**a,b**) 2 μm × 2 μm images with PFM amplitude (color) overlaid on the surface topography. The two images were taken at two different AC driving frequencies. (**c**) Frequency dependence of PFM amplitude measured at three different positions as marked in (**b**). The spatial variation of the resonance frequency (*f*_*0*_) leads to the non-uniform PFM response imaged.

At *f* = 360 kHz, PFM amplitudes overlaid as color on top of 3D surface topography are shown in Fig. 2a. Sample surfaces typically exhibited a root mean square (RMS) roughness of 3 nm. Large PFM amplitude contrast can be observed, which strongly correlates with the surface topography. Changing the AC excitation frequency to *f* = 348 kHz produces an almost opposite contrast pattern (Fig. 2b). These PFM contrasts are caused by local mechanical property variations that affect the contact resonance frequency (${f}_{0}$) of the sample-probe system^25^. As shown in Fig. 2c, PFM amplitudes measured as functions of $f$ at three positions with different local topography revealed a more than 10 kHz variation in ${f}_{0}$. Therefore, the ~100 nm scale fluctuations shown in Fig. 2a and b are topography-related and should not be confused with spontaneously formed polarization domains.

### Ferroelectric switching and built-in field

**Figure 3:**
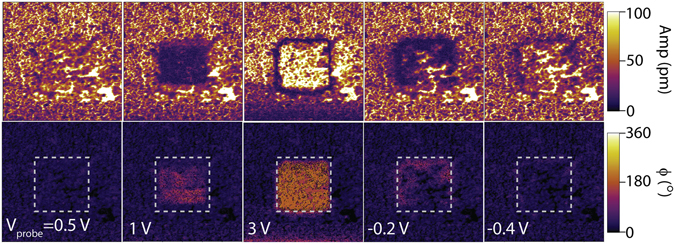
Ferroelectric switching by biased scanning probe. PFM amplitude (top) and phase (bottom) images of a 3 μm × 3 μm area consecutively taken after scanning over the center 1.5 μm square (marked with the dashed line) with different probe biases.

To investigate the ferroelectric properties, we conducted local switching experiments with biased scanning probe (Fig. 3). Starting from the as-grown film, a series of contact mode scans were performed in the same 1.5 μm square region. In each scan, a different DC probe bias (${V}_{probe}$) was applied. The changes of ${V}_{probe}$ followed the general sequence of 0 V $\to$ 10 V $\to$−10 V $\to$ 0 V. After each scan, a PFM image was taken at zero DC bias to assess the persistent polarization changes in the scanned region. Figure 3 shows five representative PFM images taken during the 0 V $\to$ 10 V ramp (0.5 V, 1 V, 3 V) and 10 V $\to$−10 V ramp (−0.2 V, −0.4 V) where a clear 180° phase shift can be seen. Other typical ferroelectric characteristics, including the minimum PFM amplitude occurring during switching and at the boundary of switched area (Fig. 3), were also observed^26–28^.

**Figure 4:**
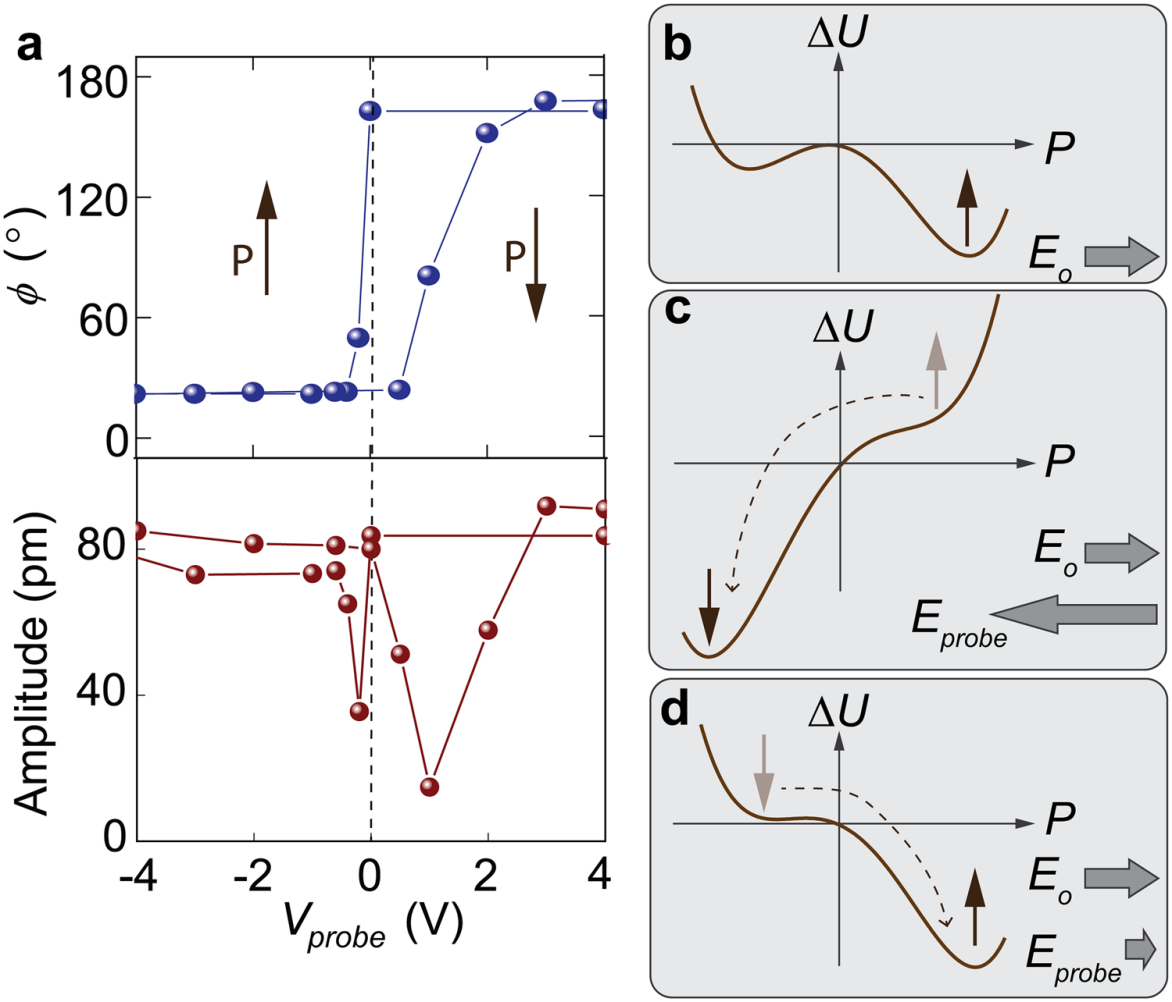
Asymmetry in ferroelectric hysteresis loop induced by built-in field. (**a**) Hysteresis of PFM phase and amplitude as a function of poling probe voltage. (**b–d**) Illustrations of the ferroelectric double well potential and polarization states affected by the built-in field (${E}_{0}$) and external probe fields.

The hysteresis curve of PFM phase ($\varphi$) versus poling bias ${V}_{probe}$ was extracted from the images by averaging the phase values of all pixels within the poled region (Fig. 4a). The averaged phase shift is less than 180° due to spatial variations of ${f}_{0}$ as discussed previously. One interesting feature of the switching loop is its highly asymmetric shape. While the positive probe bias induced switching occurred gradually between 1 V and 3 V, significant polarization reversal can be produced by negative voltages as small as −0.2 V, indicating a preferred polarization pointing out of the film (up).

It has been shown extensively that ferroelectric switching of thin films is very sensitive to the electric boundary conditions at the film surface and the interface with substrate^29–34^. Many factors, including space charge generated from band bending or charged states formed from defects/surface adsorbates, can contribute to a built-in electric field (${E}_{0}$). When the ferroelectric double well profile is shallow, the presence of a modest ${E}_{0}$ can effectively lead to a thermodynamically favored polarization direction (Fig. 4b). A large external field in the opposite direction is required to overcome *E*_0_ in order to flip the polarization state (Fig. 4c). And assisted by ${E}_{0}$, only a small external field in the same direction is needed to restore the favored polarization state (Fig. 4d). A large enough ${E}_{0}$ may even counteract the poling effect of external field completely or cause the polarization to flip back quickly afterwards, which might make the film appear unswitchable in the subsequent PFM measurements. As shown by Fig. 4a, the switch loop is almost completely shifted horizontally to the positive bias side. This indicates that the magnitude of *E*_0_ is likely very close to the coercive field. The existence of such a built-in field may critically affect the stability of the probe-poled down-polarized domains.

### Reversal of out-of-plane polarization by in-plane electric field

**Figure 5:**
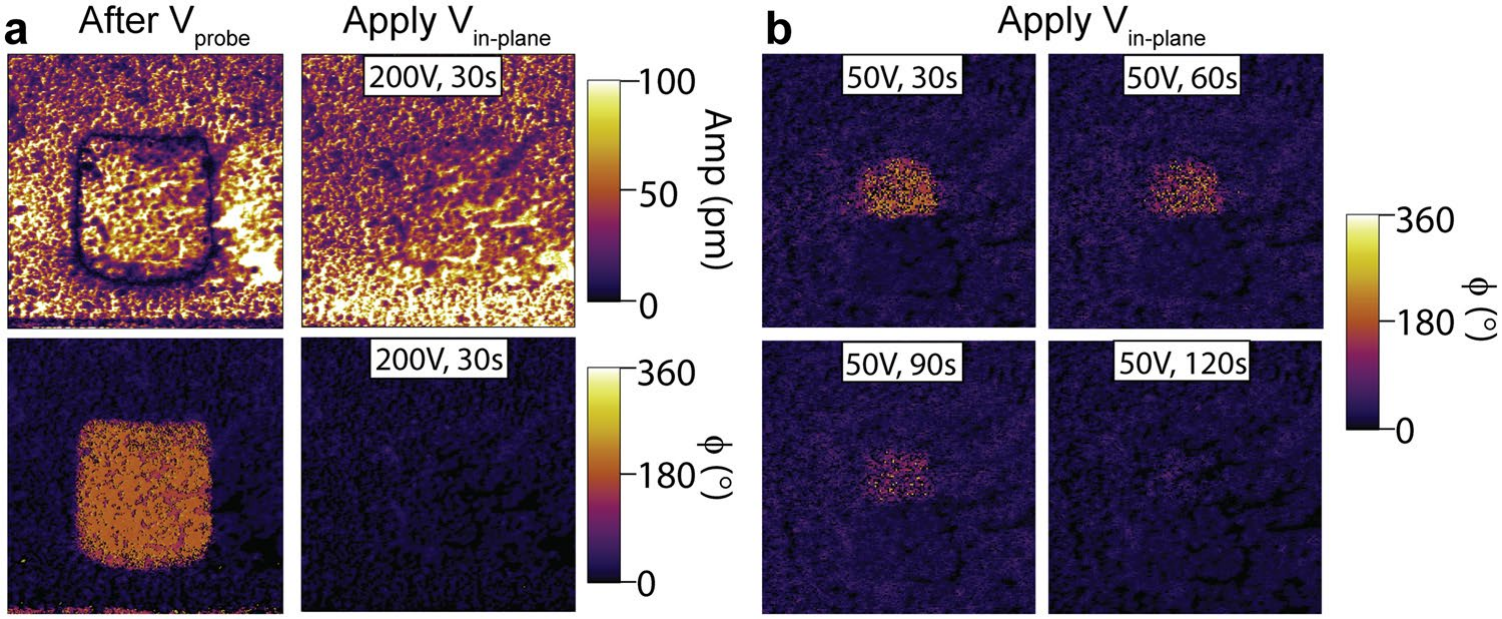
Restoration of up polarization by in-plane field. (**a**) PFM amplitude (top) and phase (bottom) measured right after the biased probe poling (left) and after the application of 200 V across the two surface contacts for a duration of 30 s (right). (**b**) PFM phase measured after consecutive applications of 30 s 50 V bias between the surface electrodes, showing the graduate reversion of the center 500 nm probe poled down-polarized region.

Unperturbed, the down polarization domain created by positive probe biases typically flips back within approximately 30 min in air. However, it was found that the application of in-plane electric field can revert these into-the-plane (down) polarized regions quickly back to the preferred up orientation. Figure 5a (left) shows the PFM images taken immediately after the center square was poled down by 10 V probe bias. This poled area was located in the middle between the two surface electrodes. After applying a 200 V relative bias across the two electrodes for 30 s, the center square region completely flipped back (Fig. 5a, right). Considering the 40 µm gap, the 200 V bias can generate an in-plane field of $5\times {10}^{6}$ V/m. The reversal of the out-of-plane polarization is slower at smaller field. At a relative electrode bias of 50 V, 120 s of in-plane field application time was needed to completely flip the down polarized domain (Fig. 5b). The observed results, however, are independent on the direction of in-plane field applied.

**Figure 6:**
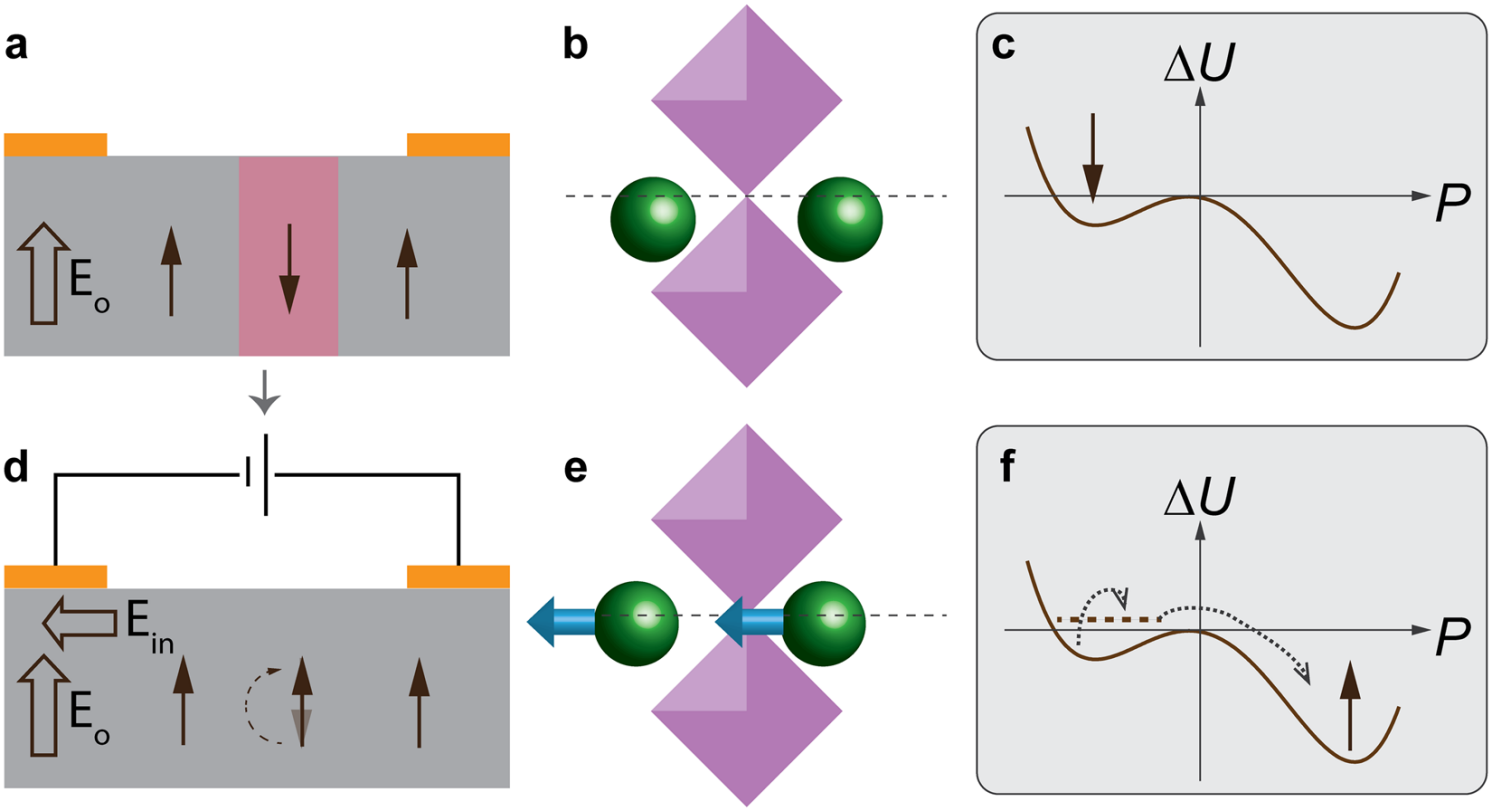
Ferroelectric switching triggered by weak perturbation. (**a,d**) Illustrations of the polarization rotation induced jointly by external in-plane field (${E}_{in}$) and the up direction built-in field (${E}_{0}$) in the region poled by positive probe bias. (**b,e**) Displacements of Na ions (green) with respect to the MnF_6_ octahedral (violet) (**b**) after probe poling and (**e**) under an external in-plane field. The octahedral tilts and rotations were removed to schematically show only the Na-site behavior, which is the one responsible for the ferroelectricity in NaMnF_3_. (**c,f**) Double well model showing (**c**) the down polarized state weakened by the built-in field (E_0_), and (**f**) polarization switching triggered by perturbation from external in-plane field.

Ferroelectric switching by biases applied between planar electrodes can be achieved when the polarization has an in-plane component^35–37^. Alternatively, cross-coupling between orthogonal electric field and ferroelectric polarization can also occur through, for example, a ferroelastic effect^38–41^. Though in that case, ferroelastic switching usually produces field-orientation-dependent polarization rotations, instead of 180° flips. In order to understand the out-of-plane polarization reversal generated by an in-plane field, regardless of the bias polarity, the effects of ${E}_{0}$ must be taken into account. As shown in Fig. 6b, Na ions are displaced downward from their high-symmetry position after being poled by the positive probe bias. However, due to the shallow ferroelectric potential well and the effect of ${E}_{0}$, the activation barrier of this down polarization state is small (Fig. 6c). When an in-plane field is applied, Na ions now move horizontally in response to the field (Fig. 6e), which produces an excitation to the ferroelectric polarization. After the external field is removed, the potential profile tilted by the built-in field then thermodynamically drives the system from the metastable perturbed state back to the favored up polarization state (Fig. 6f). Since small perturbations can be generated by Na ion displacements in any orientations in the *ac-*plane, this process also explains why the same out-of-plane polarization flip was observed independent of the in-plane field direction.

### Persistent polarization induced photocurrent

**Figure 7:**
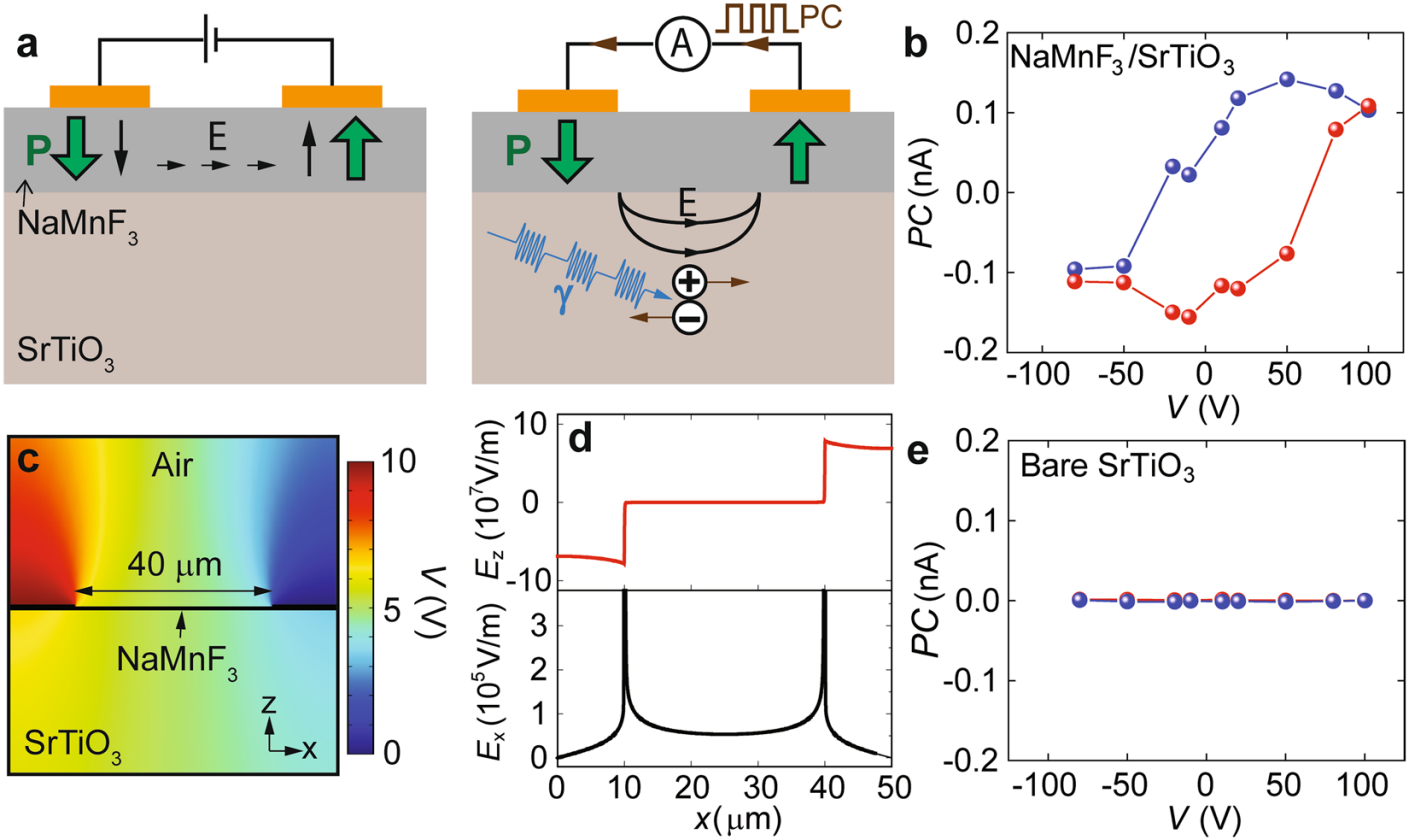
Photocurrent generated from the persistent polarizations in NaMnF_3_. (**a**) Illustration of DC bias poling and subsequent AC photocurrent measurements. (**b**) Hysteresis of AC photocurrent measured at 5 K after the application of different DC biases. Different colors indicate the data taken during increasing (red) or decreasing (blue) bias sequences. (**c**) Simulated potential distribution in the heterostructure at 10 V DC bias. (**d**) Simulated out-of-plane and in-plane field distributions in NaMnF_3_ film at 10 V DC bias. (**e**) Control experiment performed on bare SrTiO_3_ substrate under identical conditions, where no photocurrent was measured.

A field tunable zero-bias photocurrent was observed in NaMnF_3_ film samples at low temperatures. The experiment setup is illustrated in Fig. 7a. First, a DC bias was applied between the two surface electrodes with a 40 µm separation. After this DC bias was turned off, the sample was illuminated by a chopper modulated 400 nm laser beam (0.5 mW), and AC photocurrent under zero external bias was lock-in detected at the modulation frequency. Varying the pre-poling DC bias, the subsequently measured zero-bias photocurrent exhibited a clear hysteresis (Fig. 7b). As a comparison, the same experiment performed on bare SrTiO_3_ showed no measurable signal (Fig. 7e).

This observed effect can be attributed to the field poled persistent polarizations inside NaMnF_3_. As already discussed, at room temperature (RT), the application of biases up to 200 V between the surface electrodes can only generate 10^6^ V/m level field with an orientation in-plane. However, as the permittivity ($\varepsilon$) of SrTiO_3_ increases from 300 (RT) to larger than 10000 at temperatures below 20 K^42, 43^, the field profile changed dramatically (Fig. S1). With $\varepsilon  \sim 10000$, the screening effect of the substrate strongly facilitated the potential drop in the NaMnF_3_ regions underneath the electrodes, where the field became primarily out-of-plane and approached 10^8^ V/m level at a bias of 10 V (Fig. 7c,d). As a result, out-of-plane field with an intensity comparable to what was generated by nanoscale AFM probe can be produced by the surface electrodes, which switched the polarization of the two NaMnF_3_ region underneath to be opposite to each other (Fig. 7a, left).

Without external bias, the presence of two oppositely polarized regions can still generate an electric field (Fig. 7a, right). Most likely due to the defect-related in-gap states in SrTiO_3_ substrate^44–47^, electron-hole pairs can be created by the incident 3.1 eV photons. The excited electrons and holes moved in opposite directions under the influence of the polar field, leading to a photocurrent along the direction of the pre-poling DC bias. Since the direction and magnitude of such photocurrent was dependent on the orientation and strength of the persistent polarizations in NaMnF_3_, tuning of the photocurrent as shown in Fig. 7b can be achieved by controlling the pre-poling DC bias.

## Discussion and Conclusions

It is worth pointing out that one needs to be careful when interpreting PFM data in terms of ferroelectricity. This is because probe field induced PFM signal switching as presented in Fig. 3 can also be due to mechanisms other than ferroelectricity, such as hysteretic surface charging or ionic motion^48–50^. However, the also observed out-of-plane PFM signal reversal by in-plane fields applied between surface electrodes rules out these non-ferroelectric mechanisms. We note that the film remains highly insulating in our experiments. No leakage current was observed for in-plane biases up to 200 V and probe biases up to 10 V. In such an insulating state, the application of an in-plane field is not likely to deplete surface charges. Additionally, while the in-plane field might drive in-plane ion migration, in that case a distortion and drift of the probe-poled region would occur, instead of the gradual reversal observed in our experiments. In contrast, the PFM phase reversal observed can be well explained by the presence of relatively shallow ferroelectric potential wells and a built-in electric field as discussed above. Therefore, the combination of different PFM responses under the influences of out-of-plane probe field and in-plane field provided a stronger support to the ferroelectricity in NaMnF_3_ film.

As discussed above, NaMnF_3_ films grown on SrTiO_3_ substrate exhibited a preferred up-polarization because of a built-in field. This built-in field, in conjunction with the thermal activation process at room temperature, likely give rise to the short (~30 min) retention time of the down polarization state poled by positive probe biases. Consistent with the DFT model, the ferroelectricity observed is highly sensitive to small changes in growth condition. Slight variations of substrate or films thickness can produce very different material properties, as reported in ref. 17. Out of the total fourteen samples grown on different substrates and characterized by PFM, only three with films directly grown on SrTiO_3_ showed signatures of ferroelectricity. While the experiment discussed here showed signatures of theoretically predicted ferroelectricity in NaMnF_3_, the marginally stable ferroelectric polarizations are certainly not ideal. One way to enhance the ferroelectricity in NaMnF_3_ is by strain engineering^16^. Additionally, spacer and capping layers can be tailored to control the electric boundary conditions and minimize the built-in field^32, 33^. Moreover, domain walls and the coupling between in-plane and out-of-plane domains under strain can also affect the ferroelectric switching as already been theoretically investigated in the barium-based fluorides (BaZnF_4_ and BaMgF_4_)^51^. These methods and studies, as well as epitaxial quality optimizations, need to be explored to improve the stability of the polarized states and the overall ferroelectric performances.

## Methods

### Sample synthesis

NMF thin films approximately 50 nm thick were grown on pre-polished single crystal (100) SrTiO_3_ substrates (*a* = 3.905 Å) by molecular beam epitaxy (MBE) in an ultra-high vacuum chamber. During growth the pressure was 5.0 × 10^−9^ Torr. Before the growth, atomically at surface and single termination of SrTiO_3_ substrates was achieved by the combination of two thermal annealing steps and de-ionized water treatment. The films were grown via co-deposition of NaF (99.99%) and MnF2 (99.99%) using commercial Knudsen cells. The fluxes of NaF (0.027 Å/s) and MnF2 (0.043 Å/s) were measured using a quartz crystal monitor placed at the sample growth position. The growth was performed at a nominal substrate temperature of 250 °C. Growth was monitored *in-situ* using reflection high energy electron diffraction (RHEED), and x-ray diffraction and x-ray reflectivity were used to characterize the structure of the samples. More details may be found in ref. 17.

### DFT calculations

We used density functional theory (DFT) by using the projected augmented-wave method (PAW)^52^ to describe the Kohn-Sham orbitals as implemented in the Vienna *ab*-initio simulation package, VASP^53, 54^. The used electronic configurations in the PAW pseudopotentials are as follows: 7 valence electrons for Na (2p^6^3s^1^), 13 for Mn (3p^6^4s^2^3d^5^), and 7 for F (2s^2^2p^5^). The exchange correlation was represented by means of the generalized gradient approximation (GGA) with the PBEsol parameterization^55^ and corrected with the DFT + U method^56^ (U = 4.0 eV) in order to treat the localized *d* electrons of Mn within a G-type AFM ordering. This U value fairly reproduces the experimentally observed magnetic ordering and the structural properties such as vibrational modes and lattice parameters at the *Pnma* bulk ground state of the NaMnF_3_. Additionally, the qualitative results on the ferroelectric and magnetoelectric coupling remain invariant under the U and J parameters variation^16^. The structural relaxations were performed as follows: The lattice parameters that belong to the strain plain were fixed to the percentage values that takes as a reference the bulk lattice parameters. Then, the internal coordinates and the perpendicular lattice parameter to the strain plain, were allowed to relax. In this way, the volume and atomic coordinates forces were converged up to the defined tolerance value. The periodic solution was represented by using Bloch states with a Monkhorst-Pack^57^ *k*-point mesh of 8 × 6 × 8 and 700 eV energy cut-off. The latter parameters give forces converged to less than 1 meV/Å^−1^. The phonon calculations were performed with density-functional perturbation theory (DFPT)^58^, as implemented in the VASP code.

### Piezo response force microscopy (PFM)

PFM measurements were performed at room temperature using Asylum Research MFP-3D AFM system. Platinum coated Si cantilevers (Olympus OMCL-AC240TM) were used in the measurements.

### Data Availability

All data generated or analysed during this study are included in this published article (and its Supplementary Information files). Additional information are available from the corresponding author on reasonable request.

## Electronic supplementary material

Supplementary Information
