# Supplementary material for: Room temperature ferroelectricity in fluoroperovskite thin films
Source: Sci Rep. 2017 Aug 3;7:7182. doi: 10.1038/s41598-017-07834-0 (PMC5543180; doi:10.1038/s41598-017-07834-0)
Supplement: Supplementary file 1 — Supplementary Information [file 41598_2017_7834_MOESM1_ESM.pdf]

Supplementary Information: Room temperature ferroelectricity in fluoroperovskite thin films

Ming Yang[1], Amit KC[2], A. C. Garcia-Castro[1], Pavel Borisov[1,3], E. Bousquet[4], David Lederman[2], Aldo H. Romero[1], Cheng Cen[1*]

[1]*Department of Physics and Astronomy, West Virginia University, Morgantown, West Virginia 26506, USA*

[2]*Physics Department, University of California Santa Cruz, Santa Cruz, California 95064*

[3]*Department of Physics, School of Science, Loughborough University, Loughborough, LE11 3TU, UK.*

[4]*Physique Théorique des Matériaux, Université de Liège, B-4000 Sart-Tilman, Belgium*

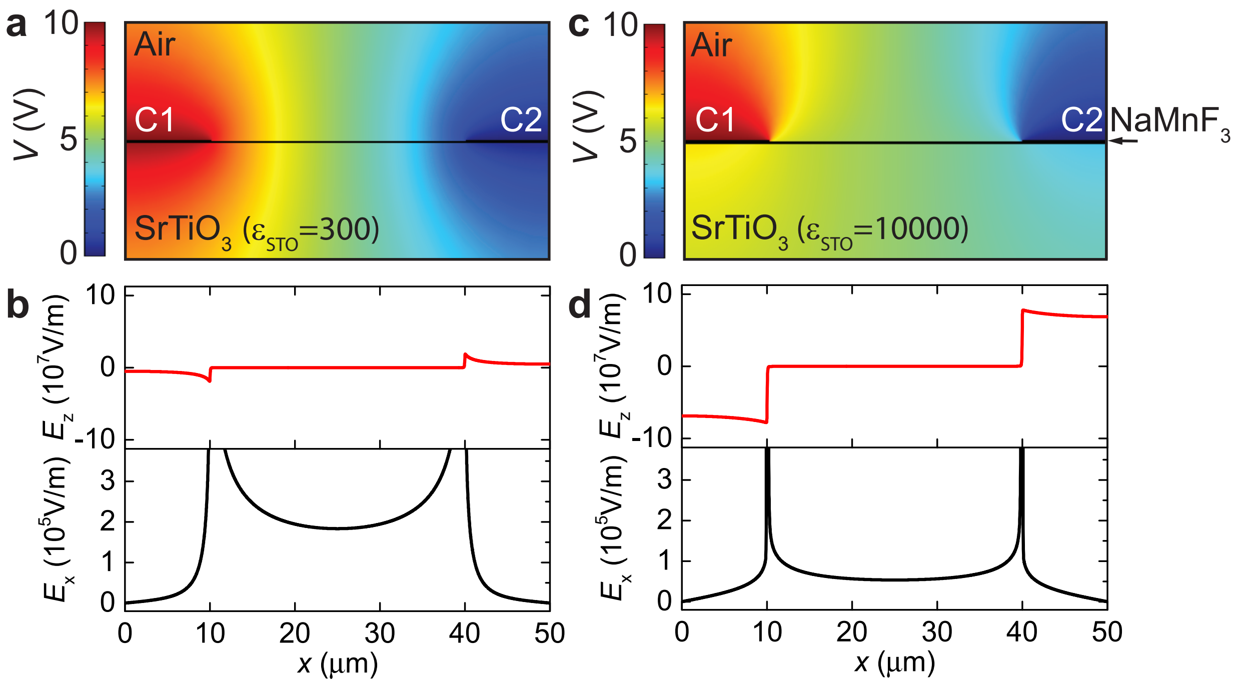

Figure S1 Finite element simulations of the field profile generated by biased surface electrodes. A bias of 10 V is applied to C1 when C2 is grounded. (a,b) Cross-sectional potential profile (a) and field variations in $NaMnF_3$ film (b) at room temperature when the dielectric constant of $SrTiO_3$ (ϵ_{STO}) is around 300. (c,d) Cross-sectional potential profile (a) and field variations in $NaMnF_3$ film (b) at low temperatures when the ϵ_{STO} gets as high as 10^4. At low temperatures, due to the strong screening of the substrate, potential drops mainly take place in the $NaMnF_3$, which leads to the large out-of-plane electric field generated.
